# Artificial Intelligence and Machine Learning: An Updated Systematic Review of Their Role in Obstetrics and Midwifery

**DOI:** 10.7759/cureus.80394

**Published:** 2025-03-11

**Authors:** Paraskevi Giaxi, Victoria Vivilaki, Angeliki Sarella, Vikentia Harizopoulou, Kleanthi Gourounti

**Affiliations:** 1 Department of Midwifery, Faculty of Health and Caring Sciences, University of West Attica, Athens, GRC

**Keywords:** artificial intelligence, machine learning, maternal, midwifery, obstetrics, pregnancy, quality improvement

## Abstract

Artificial intelligence (AI) and machine learning (ML) are rapidly evolving technologies with significant implications in obstetrics and midwifery. This systematic review aims to evaluate the latest advancements in AI and ML applications in obstetrics and midwifery. A search was conducted in three electronic databases (PubMed, Scopus, and Web of Science) for studies published between January 1, 2022, and February 20, 2025, using keywords related to AI, ML, obstetrics, and midwifery. The review adhered to Preferred Reporting Items for Systematic Reviews and Meta-Analyses (PRISMA) guidelines for updated systematic reviews. Studies were selected based on their focus on AI/ML applications in obstetrics and midwifery, while non-English publications and review studies were excluded. The review included 64 studies, highlighting significant advancements in AI and ML applications across various domains in obstetrics and midwifery. Findings indicate that AI and ML models and systems achieved high accuracy in areas, such as assisted reproduction, diagnosis (e.g., 3D/4D ultrasound and MRI), pregnancy risk assessment (e.g., preeclampsia, gestational diabetes, preterm birth), fetal monitoring, mode of birth, and perinatal outcomes (e.g., mortality rates, postpartum hemorrhage, hypertensive disorders, neonatal respiratory distress). AI and ML have significant potential in transforming obstetric and midwifery care. The great number of studies reporting significant improvements suggests that the widespread adoption of AI and ML in these fields is imminent. Interdisciplinary collaboration between clinicians, data scientists, and policymakers will be crucial in shaping the future of maternal and neonatal healthcare.

## Introduction and background

Artificial intelligence (AI) and machine learning (ML) have increasingly gained attention in healthcare, including obstetrics and midwifery, due to their potential to enhance clinical decision-making and patient outcomes. Hamet and Tremblay define AI as “the use of a computer to model intelligent behavior with minimal human intervention” [1]. Over the years, technological advancements have been closely linked to improvements in public health, and AI is expected to further contribute to better health-related outcomes, such as increased life expectancy [2]. This impact stems from AI’s ability to support scientific research, facilitate early diagnosis, and improve treatment strategies [3].

In obstetrics, AI offers numerous potential applications, particularly in diagnostic imaging and fetal monitoring. More specifically, it can be utilized in fetal cardiotocography to interpret heart rate patterns and uterine contractions more objectively, reducing observer bias and improving detection of fetal compromise. It can also be applied in ultrasonography by automating fetal biometric measurements, assist in diagnosing congenital anomalies, and enhance imaging quality. Additionally, AI can be applied in magnetic resonance imaging (MRI) for automated fetal brain and placental assessments, predicting outcomes such as the need for cerebrospinal fluid diversion in cases of ventriculomegaly and detecting placenta abnormalities with high accuracy [4].

Despite these potential benefits, some researchers are more skeptical about these benefits. Sarno et al. [5] support that there is a significant gap between the potential of AI and its current application in obstetrics. This can be attributed to several obstacles, such as the lack of standardized clinical applications. As a result, they conclude that the optimal time for widespread AI adoption in obstetrics has not yet arrived. A similar situation exists regarding the use of AI in midwifery. O'Connor et al. [6] analyzed 112 studies that examined the potential benefits of AI in midwifery, finding that only 7.14% of those studies (n=8) reported actual benefits. According to their analysis, this lack of significant effects can be attributed to poor-quality datasets that could introduce bias, the need for clinical interpretation of AI-based results, privacy and trust issues, and insufficient AI expertise among midwifery professionals.

ML refers to the improvement of computers in specific tasks by experience [7]. ML is crucial for advancing AI. More specifically, ML is the process of building or learning statistical models using previously observed real-world or simulated data to predict outcomes or categorize observations based on ‘training’ that has been provided by humans. These predictions are subsequently applied to future data, continuously incorporating new information into an ever-evolving and refined statistical model [8]. Overall, improvements in ML are considered essential in order to further advance the AI revolution [2]. The role of ML in obstetrics and midwifery has been previously studied by Barbounaki and Vivilaki [9]. This systematic review explored the applications of ML and AI in obstetrics and midwifery, analyzing 32 research studies that met methodological criteria. The authors found that ML has been successfully applied to various aspects of obstetrics, including diagnosis, pregnancy risk assessment, fetal monitoring, and reproductive medicine. Specific applications include predicting implantation outcomes in IVF, classifying sperm cells, forecasting vaginal delivery in twin pregnancies, predicting preeclampsia, and assessing neonatal mortality risks.

Since the publication of this study, there have been significant advancements in ML and AI. Over time, ML has evolved to a significant degree, driven by advances in data availability, computational power, and algorithmic innovation. By 2020, AI systems reached human parity on several benchmarks, including speech synthesis, ML, and image captioning. The General Language Understanding Evaluation benchmark emerged as a key measure of AI progress, with models achieving performance levels comparable to humans on various natural language processing tasks [10]. This progress raises significant concerns regarding the conclusions drawn from the study of Barbounaki and Vivilaki [9], which includes papers published up until 2021. Since the latest progress in ML and AI was carried out recently, it is possible that their conclusions may not fully reflect the current state of these technologies. Therefore, conducting an updated systematic review is essential to investigate the latest progress of AI and ML applications in obstetrics and midwifery.

## Review

Methods

Literature Search

The literature search of the specific study was carried out in three electronic databases (PubMed, Scopus, and Web of Science) from 01 January 2022 to 20 February 2025. This time frame was selected to focus on studies published after the publication of the paper by Barbounaki and Vivilaki [7]. As this study serves as an update to the previous review, the same keywords were used: "machine learning" AND ("intelligent systems" OR "artificial intelligence”) AND (obstetrics OR midwifery OR pregnancy OR "pregnancy risks" OR "pregnancy distress" OR "postpartum period" OR ''fetal'' OR "breast feeding" OR ''cervical''). The flow of information was carried out based on the guidelines of the Preferred Reporting Items for Systematic Reviews and Meta-Analyses (PRISMA) Statement for updated systematic reviews. The literature search was carried out using PRISMA guidelines for updated systematic reviews [10,11]. The search was carried out by the first four authors, while any potential disagreements were resolved with the help of the fifth author.

Study Selection

For the study selection, the inclusion criteria were (i) studies focusing on ML and AI and (ii) studies in the field of obstetrics and midwifery. These inclusion criteria were wide and used intentionally to encompass a wide range of studies. In addition, two exclusion criteria were applied: 1) not being published in English and 2) being a review study of any type, a meta-analysis, or an editorial. This latest criterion was not clearly mentioned in the study by Barbounaki and Vivilaki [9], although all the studies they finally included met it. Therefore, it was considered necessary to clearly mention this specific exclusion criteria in the present review. Checking the inclusion and exclusion criteria of the studies was carried out by the first four authors, while any potential disagreement was resolved with the help of the fifth author.

Data Extraction

Since this study is an update of the systematic review carried out by Barbounaki and Vivilaki [9], the same type of data was extracted from the studies. These data included (i) field of interest, (ii) problem domain in midwifery or obstetrics, (iii) authors, (iv) classifiers, (v) number of samples, and (vi) results.

Results

**Figure 1:**
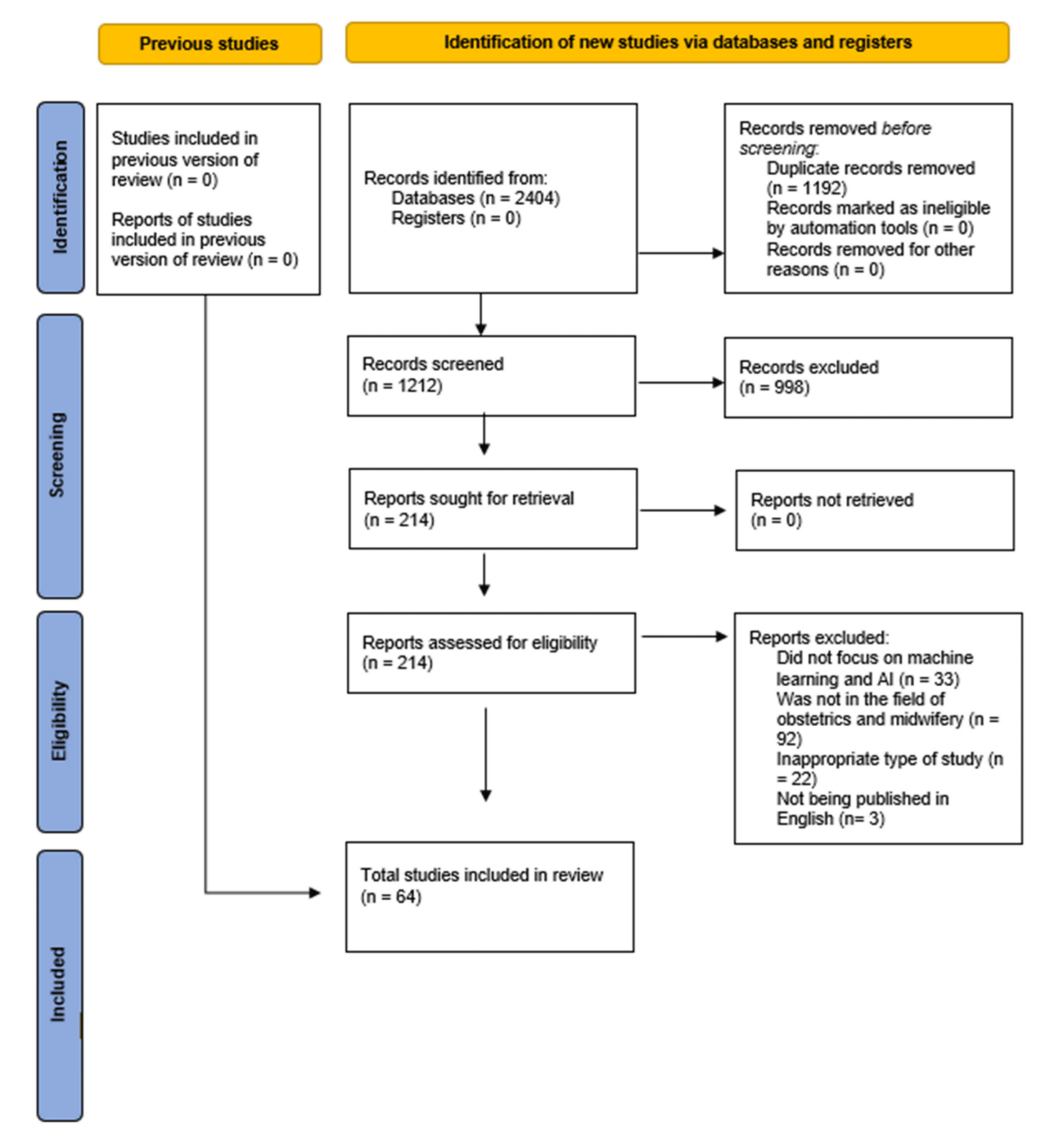
Selection process for the included studies

The search process identified 919 studies through PubMed, 772 through Scopus, and 713 through Web of Science, resulting in a total of 2404 records. No studies from the previous version of the review were included, as the search was conducted after the study of Barbounaki and Vivilaki [9]. After manually removing 1,192 duplicate records, 1,212 unique records remained. Of those, 998 were excluded based on their titles since they were obviously irrelevant to the study purpose and did not meet the inclusion criteria (e.g., they were systematic reviews). Consequently, 214 full-text articles were assessed for eligibility. Among them, 150 did not meet the necessary inclusion criteria. Therefore, 64 studies were included in the systematic review and further analyzed (Figure 1).

Embryo Selection and IVF Outcomes

The findings related to embryo selection and in vitro fertilization (IVF) outcomes are summarized in Table 1. Overall, these nine studies indicate that AI-driven models significantly enhance the ability to predict embryo viability, clinical pregnancy rates, and live birth outcomes. For instance, research by Barnes et al. [12] highlighted the effectiveness of the STORK-A algorithm in distinguishing between aneuploid and euploid embryos, achieving a notable AUC of 0.761. This model also demonstrated strong generalizability across various datasets through external validation. Similarly, Diakiw et al. [13] refined their dataset to improve the predictive accuracy of their AI model from 65.3% to 77.4%, emphasizing the critical role of high-quality data in enhancing model performance. Additionally, Ten et al. [14] utilized random forest (RF) and AdaBoost classifiers to forecast implantation success and live birth outcomes. Their findings revealed that variables such as time to hatching and cleavage pattern synchronization were pivotal in determining success, with live birth rates reported at 41.26%. Collectively, these studies illustrate how ML algorithms can enhance embryo selection processes, empowering clinicians to make better informed decisions, ultimately improving IVF efficiency, increasing success rates, and reducing patient risks.

**Table 1:** Studies Regarding Embryo Selection and IVF Outcomes

Problem Domain in Midwifery or Obstetrics	Authors	Classifiers	Number of Samples	Results
Embryo selection for transfer based on ploidy prediction	Barnes et al., 2023 [12]	Machine-learning and deep-learning algorithms (STORK-A)	10,378 embryos from 1,385 patients	STORK-A predicted aneuploid vs. euploid embryos with 69.3% accuracy (AUC 0.761), complex aneuploidy vs. euploidy and single aneuploidy with 74% accuracy (AUC 0.760), and complex aneuploidy vs. euploidy with 77.6% accuracy (AUC 0.847). External validation showed accuracies of 63.4% (AUC 0.702) on the WCM-ES+ dataset and 65.7% (AUC 0.715) on the IVI Valencia dataset, demonstrating generalisability.
Embryo prediction using static images	Diakiw et al., 2022 [13]	Artificial intelligence (AI) model	15,192 embryo images from 2,438 women	The AI model predicted euploid embryos with 65.3% accuracy (sensitivity 74.6%). After removing poor quality and mislabeled images, accuracy increased to 77.4%. Top-ranked embryos had an 82.4% chance of being euploid, increasing to 97% when considering the top two ranked embryos. The model generalized well across patient demographics and could differentiate mosaic embryos based on mosaicism levels.
Predicting implantation and live birth outcomes in egg donation IVF treatments using AI	Ten et al., 2024 [14]	Random forest, AdaBoost classification trees, multi-layer perceptron, SVM, k-nearest neighbor, CART, C0.5 classification trees, stochastic gradient boost, Bagged CART, eXtreme gradient boosting	378 egg donor recipients (fresh single embryo transfers)	Random forest had the highest predictive accuracy for implantation (AUC = 0.725, 95% CI [0.6232-0.826]), while AdaBoost was the best predictor for live birth (AUC = 0.749, 95% CI [0.6522-0.8452]). Significant variables included time to hatching (p = 0.039), duration of visible pronuclei (DESAPPN-APPN), cleavage pattern synchronization (T8-T5), duration of compaction (TM-TiCOM), and time from compaction to cavitation (TiCAV-TM). The overall implantation rate was 56.08%, the miscarriage rate was 18.39%, and the live birth rate was 41.26%.
Predicting pregnancy and multiple pregnancy risk after IVF-ET	Wen et al., 2022 [15]	XGBoost, logistic regression (LR), random forest (RF), SVM, LightGBM, multilayer perceptron (MLP)	949 IVF/ICSI cycles	XGBoost-based models achieved an AUC of 0.787 for predicting pregnancy with an accuracy of 71.6%, a sensitivity of 71.1%, and a specificity of 71.9%. For multiple pregnancy prediction, the model achieved an AUC of 0.732 with an accuracy of 71.1%. These AI models showed potential for real-time clinical use, allowing better embryo transfer decisions to reduce multiple pregnancy risk while maintaining high pregnancy rates.
Predicting clinical pregnancy rates using embryo morphokinetics with time-lapse microscopy	Yang et al., 2022 [16]	Random forest (supervised learning), unsupervised clustering	367 embryos	A supervised random forest algorithm predicted clinical pregnancy rates with 65% sensitivity, 74% positive predictive value, and an AUC of 0.7 for the test set. Unsupervised clustering grouped embryo growth morphokinetics into five clusters. Embryos with the fastest morphokinetics (time to blastocyst = 97 hours) had a pregnancy rate of 54%, while those with the slowest morphokinetics (122 hours) had a pregnancy rate of 71%. Differences between clusters were not statistically significant (P=0.356).
Prediction of fetal heart rate pregnancy across time-lapse systems	Duval et al., 2023 [17]	Hybrid model combining 3D Convolutional Neural Network (3D ConvNet) and Gradient Boosted Decision Trees (XGBoost)	9986 embryos (training and validation), 447 embryos (testing)	The hybrid model achieved an AUC of 0.727 compared to 0.684 for the video-only model. It outperformed embryologists in specificity and predictive power across different clinical contexts.
Predicting success rates in IVF	Sadegh-Zadeh et al. 2024 [18]	AdaBoost, LogitBoost, RF, SVM	495,630 records	AdaBoost Accuracy: 96.35%, Key predictors: maternal age, prior IVF cycles
Noninvasive chromosome screening for embryo grading	Li et al., 2024 [19]	Machine learning-based NICS-AI grading system	90 (NICS group) vs. 161 (Control group)	Higher clinical pregnancy rate (70% vs. 54%), ongoing pregnancy rate (58.9% vs. 44.7%), and live birth rate (56.7% vs. 42.9%), with significant benefits for women ≥35 years old
Prediction of a high proportion (>30%) of 3PN/MPN zygotes	Hong et al., 2025 [20]	AdaBoost, Gaussian NB, other ML algorithms	Data from IVF cycles (2015–2019 training set, 2019–2020 validation set)	AdaBoost was the best model; top predictors were the number of oocytes retrieved, age, male infertility, AFC, and GN stimulation days

Pregnancy Complications and Risk Prediction

The studies examining pregnancy complications and risk prediction (N=11) are summarized in Table 2. Collectively, these studies emphasize the effectiveness of AI-driven predictive models in enabling early detection and intervention for pregnancy-related conditions such as pre-eclampsia, preterm birth, gestational diabetes mellitus, and hypertensive disorders of pregnancy. For example, Hao et al. [21] demonstrated that RF models could accurately forecast adverse pregnancy outcomes among women with systemic lupus erythematosus, achieving a perfect AUC of 1.000. Key predictors identified in this model included elevated liver enzymes and reduced platelet counts. Similarly, Ansbacher-Feldman et al. [22] improved upon conventional maternal risk factor models by incorporating biomarkers such as mean arterial pressure and placental growth factor, reaching an impressive AUC of 0.909 for predicting preterm pre-eclampsia. In another significant study, Khan et al. [23] employed XGBoost alongside interpretability tools like SHAP and LIME to identify critical risk factors for preterm birth, including prior cesarean deliveries and advanced maternal age. These tools also provided individualized risk explanations, enhancing the capacity for personalized clinical decision-making. Additionally, Edvinsson et al. [24] utilized Extreme Gradient Boosting models to effectively predict ICU admissions among women with preeclampsia, relying on biochemical markers such as ASAT and uric acid, with an impressive AUC of 0.91. Taken together, these findings highlight the transformative role of AI in enhancing pregnancy care by facilitating early risk detection, promoting personalized interventions, and ultimately improving clinical outcomes for both mothers and infants.

**Table 2:** Studies Regarding Pregnancy Complications and Risk Prediction

Problem Domain in Midwifery or Obstetrics	Authors	Classifiers	Number of Samples	Results
Predicting adverse pregnancy outcomes in women with SLE	Hao et al., 2023 [21]	Random Forest, MLP, SVM, KNN, DT, LDA	51 pregnant women with 288 variables	The random forest (RF) model demonstrated the best predictive performance (AUC = 1.000), with a sensitivity of 81.3% and a specificity of 89.5%. Real-time models showed high discriminative abilities, particularly when developed using variables from pre-pregnancy to the second trimester (AUC = 0.982). Variables such as ALT, GGT, ANA titer, TT, and platelet count were identified as significant predictors for adverse pregnancy outcomes.
Predicting pre-eclampsia using first-trimester biomarkers	Ansbacheret-Feldman al., 2022 [22]	Artificial Neural Network (ANN)	60,789 pregnancies	The model achieved an AUC of 0.909 for predicting preterm pre-eclampsia (PE) with a 10% false-positive rate (FPR) and a detection rate of 75.3%. Without race information, detection rates decreased significantly (55-63% depending on race). Key biomarkers included mean arterial pressure (MAP), uterine artery pulsatility index (UtA-PI), and placental growth factor (PlGF). Incorporating these biomarkers improved predictive accuracy compared to maternal factors alone.
Predicting preterm birth and identifying key risk factors in pregnant women	Khan et al., 2023 [23]	XGBoost, SHAP (SHapley Additive exPlanations), LIME (Local Interpretable Model-agnostic Explanations)	3,509 pregnant women in the UAE	The XGBoost model achieved an AUC of 0.735 for multiparous women and 0.723 for nulliparous women. The overall PTB incidence was 11.23%. SHAP and LIME analyses provided individualized risk explanations, enhancing clinical interpretability and supporting targeted care interventions to reduce PTB-related morbidity and mortality.
Predicting severe outcomes and need for ICU admission in women with preeclampsia	Edvinsson et al., 2024 [24]	XGBoost (Extreme Gradient Boosting)	81 (41 ICU, 40 control)	The best model using ASAT, uric acid, and BMI achieved an AUC of 0.91 (cross-validation) and 0.85 (test set); accuracy was 0.88 (cross-validation) and 0.82 (test set).
First-trimester screening for pre-eclampsia in an Asian population	Nguyen‐Hoang et al., 2024 [25]	AI, ML, FMF competing risk model	10,935 singleton pregnancies	Model 1 (AI+ML model derived from UK data) achieved AUCs of 0.82 (preterm pre-eclampsia), 0.75 (term pre-eclampsia), and 0.78 (any pre-eclampsia), all significantly lower than the FMF competing risk model. Model 2, adjusted for PlGF analyzers, improved AUCs to 0.84, 0.77, and 0.80 respectively, achieving comparable performance to the FMF model for preterm and term pre-eclampsia but slightly lower performance for predicting any pre-eclampsia.
Identifying clinical and dental predictors of preterm birth (PTB) and spontaneous PTB (SPTB)	Park et al., 2024 [26]	Random forest (RF), SHAP (Shapley Additive Explanations)	60 women (30 PTB, 30 full-term birth)	The random forest model identified the top predictors of PTB as pre-pregnancy BMI, modified gingival index (MGI), preeclampsia, decayed missing filled teeth (DMFT) index, and maternal age. SHAP analysis showed positive correlations between PTB/SPTB and predictors such as premature rupture of membranes, pre-pregnancy BMI, maternal age, and MGI. These findings emphasize the importance of integrated medical and dental care during pregnancy. Future research should focus on larger populations and interventions to mitigate risk factors.
Early detection of pre-eclampsia in pregnancy	Gil et al., 2024 [27]	Fully connected neural network (machine learning model)	10,110 pregnancies	Detection rate at 10% screen-positive rate (SPR): Early PE 84.4%; preterm PE 77.8%, All PE 55.7%; area under the curve (AUC): Early PE 0.920, Preterm PE 0.913, All PE 0.846
Predicting LGA in women with gestational diabetes mellitus (GDM)	Kang et al., 2024 [28]	Convolutional Neural Network (CNN)-based fusion model	371 women with GDM	The AI fusion model achieved an AUCROC of 0.852 (95% CI 0.680-0.966) and an accuracy of 84.4%. The model outperformed three conventional models by using 24-hour CGM data and 15 clinical variables to predict LGA risk effectively.
Predicting the risk of GDM in early pregnancy	Li et al., 2024 [29]	XGBoost, Logistic Regression, Ensemble Algorithms	4799 (XHCM cohort), 2795 (SPNPH cohort)	XGBoost achieved AUC = 0.75 (XHCM initiation), 0.99 (end of first trimester), and 0.83 in SPNPH. Key predictive factors: pre-pregnancy BMI, abdominal circumference, FPG, and HbA1c.
Early prediction of hypertensive disorders during pregnancy using lifestyle data	Mizuno et al., 2024 [30]	Logistic Regression, Random Forest, XGBoost	23,000 pregnancies	The best model achieved AUC = 0.93. Key predictive features included eating habits and comprehensive lifestyle factors, enabling early detection for timely interventions.
Early detection of preeclampsia based on sociodemographic and obstetric factors	Kaya et al., 2024 [31]	Extreme Gradient Boosting (XGB) and other ML algorithms	100 mothers (22 nulliparous with preeclampsia, 25 nulliparous without, 28 multiparous with, 25 multiparous without)	XGB classifier had the highest accuracy (70% and 72.7%); maternal BMI and family history of diabetes were key predictors

Maternal Health and Morbidity

The studies addressing maternal health and morbidity (N=29) are summarized in Table 3. Overall, these studies demonstrate how predictive algorithms can effectively assess the risk of adverse outcomes, including postpartum hemorrhage, hypertensive disorders, gestational diabetes, and severe maternal morbidity. For example, Arora et al. [32] applied deep learning techniques to analyze placental ultrasound images across various trimesters, with the EfficientNetB0 model achieving a notable accuracy rate of 87.5% and high specificity for predicting adverse maternal outcomes. In another important study, Tesfa et al. [33] utilized RF and other ML algorithms to forecast pregnancy termination risks among adolescents. Their analysis identified marital status, age, and educational attainment as key predictive factors, achieving a strong AUC of 0.93. Similarly, Susanu et al. [34] developed a Naïve Bayes model specifically designed to predict the likelihood of postpartum hemorrhage, which achieved a high sensitivity rate of 96.3%, surpassing other algorithms in its ability to detect both mild and severe hemorrhage cases effectively. Collectively, these findings highlight the significant potential of AI-driven models to transform maternal healthcare by enabling early identification of high-risk conditions. These models support personalized interventions and enhance healthcare providers' ability to manage complex pregnancies more effectively, ultimately contributing to improved maternal health outcomes.

**Table 3:** Studies Regarding Maternal Health and Morbidity

Problem Domain in Midwifery or Obstetrics	Authors	Classifiers	Number of Samples	Results
Classification of placental ultrasound images across trimesters and prediction of adverse materno-fetal outcomes	Arora et al., 2023 [32]	Deep Learning, Transfer Learning, Vision Transformer, Inception v3, EfficientNetB0	1,008 cases (600 normal outcomes)	The Inception v3 model achieved an accuracy of 83.3% and a Cohen’s kappa of 0.662 when classifying between T1 and T2 images. The EfficientNetB0 model achieved 87.5% accuracy, a Cohen’s kappa of 0.749, a sensitivity of 83.4%, and a specificity of 88.9% when classifying T1 and T3 images. For predicting adverse outcomes, the F1 scores were 0.824 (T1), 0.820 (T2), and 0.892 (T3). Sensitivity and specificity increased from 77.4% and 80.2% at T1 to 81.0% and 93.9% in later trimesters.
Predicting pregnancy termination among adolescents	Tesfa et al., 2024 [33]	Random forest, Decision Tree, Logistic Regression, SVM, XGBoost, AdaBoost, CatBoost, KNN, Feedforward Neural Network	75,210	The random forest (RF) model had the highest performance with an accuracy of 92.9% and an AUC of 0.93. Significant predictors included marital status, age, parity, and educational attainment.
Predicting intra/postpartum hemorrhage	Susanu et al., 2024 [34]	Naïve Bayes (NB), Decision Tree (DT), Random Forest (RF), Support Vector Machine (SVM)	203 patients	The Naïve Bayes (NB) algorithm showed the highest accuracy (98.6%) and sensitivity (96.3%) for predicting PPH, with a false negative rate of 3.7%. The decision tree (DT) and RF algorithms also had high sensitivities (>94%). For mild hemorrhage classification, NB and SVM had superior sensitivity (96.4%) and accuracy (92.1%). Severe cases were best predicted by NB (sensitivity 89.3%, accuracy 82.4%). Overall, NB and SVM outperformed other algorithms in predicting both mild and severe hemorrhage.
Predicting optimal timing for intrauterine insemination and timed intercourse	Youngster al., 2023 [35]	NGBoost ML, treatment management algorithm	2,467 cycles	The model predicted optimal timing for IUI and timed intercourse with an accuracy of 92.9% (expert test set) and 92.4% (certain ovulation test set). Error rates were 4.2% and 3.1%, respectively. The most influential features included estradiol, progesterone, and luteinizing hormone levels. The model effectively minimized missed ovulation events and errors, outperforming conventional methods and showing promise for improved ovulation prediction and IUI scheduling.
Predicting pregnancy complications in women undergoing ART	Wang et al., 2024 [36]	Logistic regression (LR), RF, gradient boosting (GB)	14,732 women undergoing ART cycles	Prediction models showed C-statistics of 66%-75% for predicting preeclampsia, placental complications, and postpartum hemorrhage. The most influential predictors included age, BMI, infertility diagnosis, and the type of embryo transfer (fresh or frozen). Inclusion of treatment-related variables slightly improved prediction performance by 1%-5%. Logistic regression consistently performed comparably to more complex models like random forests and Gradient Boosting.
Early prediction of PCOS using EHR data	Zad et al., 2024 [37]	Logistic regression, SVM, gradient boosted trees, random forest, neural network (MLP)	30,601 women aged 18-45	Machine learning models achieved an average AUC of 85%, 81%, 80%, and 82% across four models for predicting PCOS based on EHR data. Significant positive predictors included hormone levels (FSH, LH, estradiol, SHBG) and obesity, while negative predictors were gravidity and positive bHCG. The model has potential for early detection and integration into EHR systems for timely PCOS diagnosis and intervention.
Predicting hypertensive disorders and gestational diabetes in twin pregnancies	Mustafa et al., 2024 [38]	Logistic Regression, XGBoost (ML)	707,198 for HDP, 723,882 for GDM	The incidence of hypertensive disorders in pregnancy (HDP) increased from 12.2% (2016) to 15.4% (2021), and gestational diabetes mellitus (GDM) increased from 8.1% (2016) to 10.7% (2021). Predictors for HDP included maternal age (<20 or ≥35), infertility, obesity, non-Hispanic Black race, and Medicaid insurance. GDM predictors included maternal age (≥30), non-Hispanic Asian race, non-US nativity, and obesity. The AUC for ML models was 0.62±0.004 (HDP) and 0.67±0.004 (GDM), showing similar performance to logistic regression models.
Predicting adverse maternal and fetal outcomes in preeclampsia	Zheng et al., 2023 [39]	Logistic regression, random forest, multi-layer perceptron (MLP), support vector machine (SVM), Decision Tree (DT)	733 women diagnosed with preeclampsia	The random forest, MLP, and SVM models demonstrated superior discriminative power with higher AUC scores. Decision tree, random forest, and logistic regression models showed better calibration. K-Nearest Neighbor was the most accurate imputation method, and the imputation process did not significantly affect model performance. Machine learning models had better prediction accuracy, while logistic regression showed superior calibration for outcome predictions. Larger datasets are needed for stronger evidence.
Predicting biochemical pregnancy loss after transfer of euploid embryos from PGT-A cycles	Oritiz et al., 2024 [40]	Random Forest, SHAP (SHapley Additive exPlanations), and four other supervised ML algorithms	6,020 embryos from 2,879 PGT-A cycles (1,161 single embryo transfers)	The RF model achieved the best performance with an AUC of 0.913, an accuracy of 0.830, a positive predictive value of 0.857, and a negative predictive value of 0.807. SHAP analysis revealed that embryo biopsy variables had the highest predictive power, followed by ovarian stimulation factors (number of oocytes retrieved, duration of stimulation), maternal age, and paternal age. The model effectively predicted BPL occurrences after euploid embryo transfer.
Predicting pregnancy termination and identifying associated factors among reproductive-aged women in East Africa	Setegn & Dejene, 2024 [41]	Bagging Classifier, Random Forest, Extreme Gradient Boosting (XGB), CatBoost Classifier, Extra Trees Classifier, SHAP, Eli5, LIME (XAI methods)	338,904 instances from DHS data across six East African countries	RF achieved the highest prediction accuracy of 85.6%. Other models had accuracies ranging from 79.4% to 85.6%. XAI techniques (SHAP, Eli5, LIME) identified wealth index, current working experience, source of drinking water, sex of household head, education level, and marital status as the most significant factors associated with pregnancy termination. The study's findings can help healthcare providers in East Africa target interventions for populations at higher risk.
Predicting gestational age using fetal ultrasonography images and videos	Lee et al., 2023 [42]	AI-based image model, video model, ensemble model (combining image and video models)	3,842 participants (404 in the test set)	The ensemble model achieved the lowest mean absolute error compared to standard fetal biometry-based estimates (mean difference: -1.51 [3.96] days; 95% CI, -1.90 to -1.10 days). All models (image, video, and ensemble) statistically outperformed traditional biometry estimates. The AI models were particularly accurate in fetuses predicted to be small for their GA, demonstrating potential for improving prenatal care by enabling more accurate and reliable GA assessments by trained operators.
Identifying clinical variables predicting SARS-CoV-2 infection in pregnant and non-pregnant women	Futterman et al., 2022 [43]	Safe people artificial intelligence (SPAI) platform	1,935 non-pregnant women (positive), 1,909 non-pregnant women (negative); 280 pregnant women (positive), 1,000 pregnant women (negative)	The most critical clinical variable for predicting a positive SARS-CoV-2 test in non-pregnant women was age. In pregnant women, elevated D-dimer levels and abnormal fetal heart rate patterns were the most predictive variables. The SPAI platform identified distinct clinical factors for pregnant and non-pregnant populations, offering insights into risk stratification and supporting individualized patient care strategies for SARS-CoV-2 infection.
Developing a screening method for spontaneous preterm birth using AI	Andrade Júnior et al., 2023 [44]	Stacking-based ensemble learning method (SBELM), neural network, logistic regression (LR)	524 singleton pregnancies (18th-24th week gestation)	The SBELM model outperformed cervical length (CL < 25 mm) measurement alone with an AUC of 0.808 compared to 0.318, a sensitivity of 47.3% vs. 33.3%, a specificity of 92.8% vs. 91.8%, a positive predictive value of 32.7% vs. 23.1%, and a negative predictive value of 96.0% vs. 94.9%. The model demonstrated statistical significance (p < .00001) and improved screening performance for sPTB < 35 weeks, offering a viable alternative with a low false-positive rate compared to traditional methods.
Predicting the occurrence and severity of intrahepatic cholestasis of pregnancy (ICP) using AI	Ren et al., 2025 [45]	Random forest, Support Vector Machine, Artificial Neural Network, CatBoost (Best Model)	798 participants (300 normal, 312 mild, 186 severe cases)	The CatBoost model achieved the highest predictive performance with an AUC of 0.9614 (95% CI [0.9377-0.9813]), an accuracy of 90.85%, a precision of 89.30%, a recall of 90.59%, and an F1-score of 89.81%. Eleven critical risk factors were identified, including total bile acid levels, gamma-glutamyl transferase, multiple pregnancy status, lymphocyte percentage, hematocrit, neutrophil percentage, prothrombin time, aspartate aminotransferase, red blood cell count, lymphocyte count, and platelet count.
Developing and validating an AI model for movement recognition in full-term and preterm infants	Lin et al., 2024 [46]	17-point Human Pose Estimation Model, Skeleton-Based Action Recognition Model	84 infants (30 full-term, 54 preterm) contributing 13,139 video samples	The AI algorithm accurately classified 31 selected movements from the Alberta Infant Motor Scale, achieving an accuracy, recall, precision, and F1 score of 0.91. High intra- and inter-rater reliability (88%-100%) was observed among pediatric physical therapists in annotating the videos. This model demonstrated robust potential for future remote infant movement assessments through home video recordings.
Predicting GDM using early pregnancy placental ultrasound imaging with ML and clinical features	Zhou et al., 2024 [47]	RBF-SVM, deep convolutional neural network (DLCNN), ResNet-50, Nomogram	415 pregnant women (305 discovery cohort, 110 validation cohort)	The ML model achieved AUCs of 0.91 (discovery) and 0.86 (validation). DLCNN showed lower AUCs of 0.65 and 0.69, while the clinical model had AUCs of 0.66 for both cohorts. The nomogram had the highest AUCs of 0.93 and 0.88. Calibration curves indicated excellent model fit and decision curve analysis (DCA) demonstrated superior clinical utility.
Predicting exclusive breastfeeding during the in-hospital postpartum stay using ML and XAI	Oliver-Roig et al., 2022 [48]	XGBoost with explainable AI (XAI) using SHAP values	2042 mothers across 18 hospitals in Eastern Spain	The XGBoost model achieved a ROC AUC of 0.78, PR AUC of 0.86, accuracy of 0.75, and a Brier score of 0.17. Key predictors included pacifier use, breastfeeding self-efficacy, previous breastfeeding experience, birth weight, neonatal care unit admission, timing of first skin-to-skin contact, and Baby-Friendly Hospital Initiative accreditation. The model captured non-linear relationships and individual risk variations, supporting targeted care improvements.
Predicting the optimal day of trigger during ovarian stimulation	Fanton et al., 2022 [49]	Linear regression, follicle imputation, trigger-day recommendation algorithm	30,278 cycles	Early triggers resulted in 2.3 fewer mature oocytes (MII), 1.8 fewer fertilized oocytes (2PNs), and 1.0 fewer usable blastocysts compared to on-time triggers; late triggers resulted in 2.7 fewer MII, 2.0 fewer 2PNs, and 0.7 fewer usable blastocysts. The model improves outcomes by optimizing the trigger day.
Accurate identification of embryos during cleavage and blastocyst stages	Hammer et al., 2022 [50]	Convolutional neural network (CNN)	4889 embryo images (400 patient cohorts for testing)	The CNN model achieved 100% accuracy in identifying embryos across random pools of eight patient embryo cohorts on both day 3 (~70 hpi) and day 5 (~110 hpi). The algorithm generated unique identification keys based on morphological features, offering a robust and automated specimen tracking system for embryology laboratories.
Prediction of gestational diabetes using large cohort data	Watanabe et al. (2023) [51]	Gradient Boosting Decision Tree (GBDT), Random Forest, SVM	82,698	GBDT achieved highest accuracy, AUC 0.74 for GDM without prior history.
Predicting risk of FAS among pregnant women who consumed alcohol	Oh et al., 2023 [52]	CatBoost, XGBoost, Light Gradient Boosting Machine, Logistic Regression	595 women (20 FAS cases)	The CatBoost model had the highest AUROC (0.92), AUPRC (0.51), and an accuracy of 0.96. Important predictors included alcohol consumption throughout pregnancy, maternal age, race, and type of alcoholic beverage consumed. Boosting methods improved prediction for imbalanced data.
Pregnancy prediction post-IVF treatments	Cao et al., 2025 [53]	Gradient Boosting Machine (GBM), SHAP analysis	2865 couples	The GPT-4 model achieved an accuracy of 0.79 and AUROC of 0.89, surpassing the previous study's metrics. Identified key predictors: female age, progesterone level, fasting glucose, sperm motility, and sperm density.
Predicting postpartum hemoglobin levels to identify hemorrhage risk	Aghajanian et al., 2024 [54]	Elastic Net Regression, Random Forest, Artificial Neural Network (ANN)	1974	The ANN achieved highest accuracy with RMSE = 0.62. Key predictors: parity, gestational age, pre-delivery hemoglobin, fibrinogen levels, and platelet count. Developed as a web-based tool.
Predicting risk factors for episiotomy during vaginal delivery	Banaei et al., 2024 [55]	Linear Regression, Deep Learning, SVM, LightGBM, Logistic Regression, XGBoost, Random Forest, Decision Tree, KNN	1775	Linear regression performed best (AUC = 0.85, accuracy = 0.80). Top predictors: parity, labor onset, gestational age, BMI, and doula support.
Identifying risk factors for postpartum depression using antepartum data	Wakefield & Frasch, 2023 [56]	Distributed Random Forest, Logistic Regression	8,454	Best-performing model (AUC = 0.91). Top predictors: history of depression, mental health condition, psychiatric medication use, BMI, income, age, anxiety history.
Classifying fetal health status using Cardiotocography (CTG) data	Mushtaq & Veningston, 2024 [57]	Deep Neural Network (DNN), Logistic Regression, KNN, SVM, Naive Bayes, Random Forest, Gradient Boosting	Not specified	DNN: Accuracy 0.99, sensitivity 0.93, specificity 0.93, AUC 0.96, precision 0.93, F1 score 0.93; outperformed all baseline models (highest baseline accuracy: 0.93)
Risk prediction for ICU admission among pregnant women with severe maternal morbidity	Soares et al., 2024 [58]	Decision Trees, Random Forest, Gradient Boosting Machine (GBM), Extreme Gradient Boosting (XGBoost)	9550 pregnant women	XGBoost showed the best performance with an accuracy of 85%, a sensitivity of 42%, a specificity of 97%, and an AUC of 86.7%. The model-estimated ICU utilization rate was 11.6%, compared to the observed rate of 21.52%, highlighting the need for optimizing ICU bed allocation for high-risk pregnancies.
Prediction of Severe Maternal Morbidity (SMM)	Clapp et al., 2022 [59]	Natural Language Processing (NLP) vs. Obstetric Comorbidity Index (OB-CMI)	19,794 patients	NLP AUC = 0.76, OB-CMI AUC = 0.74; Combined Model Improved Sensitivity to 37.4%
Identifying psychosocially vulnerable groups among parenting mothers	Hanai et al. (2024) [60]	Explainable k-means clustering	1559 mothers (310 newborns, 619 infants, 461 toddlers)	The classifier stratified participants into five groups based on resilience and adaptation. The most vulnerable groups showed higher incidences of depressed mood (RP = 5.87–9.05), bonding issues (RP = 1.63–5.38), and sleep disturbances (RP = 1.70–8.69).

Studies Regarding Fetal Health and Development

The five studies focusing on fetal health and development are summarized in Table 4, which collectively emphasize the significant potential of AI and ML in improving prenatal care through early detection of fetal abnormalities, enhancing diagnostic accuracy, and supporting clinical decision-making. These studies highlight the effectiveness of AI algorithms in analyzing various data sources, including ultrasound images, MRI scans, and electronic fetal monitoring (EFM) records, to assess fetal conditions accurately. For example, Ghi et al. [61] developed a neural network model that successfully classified fetal head positions during labor with an impressive accuracy rate of 90.4%, providing valuable real-time support for obstetricians and midwives during labor management. In a similar vein, Bachnas et al. [62] utilized convolutional neural networks (CNNs) applied to 3D/4D ultrasound imaging to detect facial abnormalities, including cleft lip and Down syndrome, with high accuracy and specificity, significantly improving the potential for early diagnosis. Additionally, Vasung et al. [63] developed a 3D CNN model to analyze fetal limb movements, uncovering developmental patterns that could serve as early biomarkers for fetal health and development. Collectively, these studies underscore the transformative role of AI in fetal monitoring by enabling early interventions, enhancing diagnostic precision, and facilitating personalized care strategies. These advancements contribute to improved perinatal care quality and better health outcomes.

**Table 4:** Studies Regarding Fetal Health and Development

Problem Domain in Midwifery or Obstetrics	Authors	Classifiers	Number of Samples	Results
Automatic recognition of fetal head position using transperineal ultrasound during labor	Ghi et al., 2022 [61]	Pattern-recognition feed-forward neural network	1,219 women (801 OA, 418 non-OA)	The ML algorithm correctly classified fetal head positions in 90.4% of the test dataset. The model accurately identified 91.1% of occiput anterior (OA) positions and 89.3% of non-OA positions. It achieved an F1-score of 88.7% and a precision-recall area under the curve (PR-AUC) of 85.4%. Cohen’s kappa was 0.81 (P < 0.0001), indicating high agreement with the gold standard transabdominal ultrasound classification.
Early detection and diagnosis of fetal facial abnormalities	Bachnas et al., 2024 [62]	Convolutional neural networks (CNNs), AI-enhanced 3D/4D ultrasound	1219 women	The AI model improved detection of fetal facial abnormalities, including cleft lip, Down syndrome, and craniofacial syndromes, achieving high accuracy and specificity.
Quantifying 3D fetal limb movements using MRI and ML	Vasung et al., 2023 [63]	3D convolutional Neural Network (CNN) for fetal keypoint tracking	76 MRI scans from 52 women (24–40 weeks gestation)	Machine learning quantified fetal limb movements, revealing longer movement durations during maternal hyperoxia. The study suggests potential biomarkers for fetal health.
Improving detection accuracy of CHD during second-trimester ultrasound scans	Athalye et al., 2024 [64]	Deep learning (DL) model	108 (66 CHD cases, 42 normal fetuses)	Model sensitivity: 91%, specificity: 78%; human experts' mean sensitivity: 55% (range 47-67%), specificity: 71% (range 57-83%); DL model outperformed experts on unseen lesions (16/19 correct); Image quality affected model accuracy (P = 0.03); model sensitivity exceeded initial clinical assessments (53%)
Predicting fetal acidemia using electronic fetal monitoring (EFM) data	McCoy et al., 2025 [65]	Deep learning architectures (unspecified)	10,182 EFM recordings	The best model achieved an AUC of 0.85 for predicting pH < 7.05. For predicting both pH < 7.05 and base excess < -10 meq/L, the AUC was 0.89. At pH < 7.15 with a positive predictive value of 30%, the model achieved 90% sensitivity and 48% specificity. External validation confirmed model performance.

Labor and Delivery Outcomes

The studies on labor and delivery outcomes (N=4), summarized in Table 5, highlight the crucial role of ML and AI in predicting childbirth complications and optimizing clinical decision-making during labor. These studies primarily focus on improving the accuracy of predicting adverse outcomes such as unplanned cesarean deliveries, postpartum hemorrhage, and neonatal morbidity by leveraging real-time data and advanced predictive algorithms. For example, Meyer et al. [66] developed an XGBoost model that demonstrated high predictive accuracy, achieving AUCs of up to 0.874 for forecasting unplanned cesarean deliveries. This model effectively identified high-risk subgroups across various gestational ages, allowing for timely clinical interventions. Similarly, Shazly et al. [67] created a gradient boosting model that dynamically assessed labor progression using intrapartum clinical data, with its predictive accuracy improving from an AUC of 0.75 to 0.89 as cervical dilation advanced. This model offered personalized risk scores for complications such as postpartum hemorrhage and neonatal morbidity. Another significant contribution by Lipschuetz et al. [68] involved the use of electronic health records to predict labor outcomes, with real-time data inputs substantially improving model accuracy, particularly in predicting the likelihood of a successful vaginal delivery after a previous cesarean section. Collectively, these studies underscore the transformative potential of AI-driven predictive models in providing dynamic, personalized risk assessments that support clinical decision-making and reduce complications for both mothers and newborns during labor and delivery.

**Table 5:** Studies Regarding Labor and Delivery Outcomes

Problem Domain in Midwifery or Obstetrics	Authors	Classifiers	Number of Samples	Results
Predicting unplanned cesarean delivery among singleton pregnancies	Meyer et al., 2023 [66]	Random Forest for feature selection, XGBoost for prediction	73,667 women (training: 80%, validation: 20%, test: separate cohort)	The XGBoost model achieved AUCs of 0.874 (training), 0.839 (validation), and 0.840 (test set). The model included 13 features and had a positive predictive value (PPV) of 65% for women in the 100th centile group and a negative predictive value (NPV) of over 99% in women below the 50th centile. High predictive accuracy was maintained across gestational ages 34–42 weeks and among high-risk subgroups.
Predicting unfavorable labor outcomes using intrapartum clinical data	Shazly et al., 2022 [67]	Gradient Boosting Algorithm	66,586 deliveries from a total of 228,438 episodes (Consortium on Safe Labor database)	The model predicted a composite of adverse maternal and neonatal outcomes. The AUC improved from 0.75 (at 4 cm cervical dilation) to 0.89 (at 10 cm cervical dilation). The baseline labor risk score exceeded 35% in women with unfavorable outcomes while remaining below 25% for those with favorable outcomes. The machine-learning-based labor risk score offers dynamic and individualized risk assessments for clinical decision-making during labor progression.
Identifying high-risk obstetric patients before and during labor using electronic health records	Lipschuetz et al., 2024 [68]	Machine Learning (ML), Real-time dynamic models	130,000 births (~180 million data points)	Machine learning models accurately predicted successful vaginal deliveries both in the general population and among women attempting trial of labor after cesarean delivery. Prediction accuracy improved as more real-time data became available during labor. An AI model developed for cross-facility prediction of unplanned cesarean deliveries highlighted challenges related to variations in reporting practices across institutions. The models aimed to personalize birth management and reduce complications for mothers and newborns globally.
Postpartum hemorrhage prediction after cesarean section	Dogru et al., 2025 [69]	Logistic Regression (LR) with Class Weights, Support Vector Machine (SVM), Random Forest (RF), Multilayer Perceptron (MLP)	615 twin pregnancies (150 with PPH, 465 without PPH)	LR with class weights had the highest AUC (75.12%), accuracy (70.73%), PPV (47.92%), and NPV (85.33%)

Neonatal Outcomes and Mortality

The studies on neonatal outcomes and mortality (N=6), presented in Table 6, underscore the significant potential of ML and AI in predicting adverse neonatal conditions, improving early diagnosis, and guiding clinical interventions. These studies collectively demonstrate how predictive algorithms can assess key risk factors associated with respiratory distress syndrome (RDS), low birth-weight complications, and neonatal mortality. For example, Sridharan et al. [70] applied the C5.0 algorithm alongside Bayesian networks to identify genetic markers and oxidative stress biomarkers linked to neonatal RDS and liver function alterations, achieving moderate predictive performance with AUCs of 0.63 and 0.60, respectively. Overall, the studies in Table 6 highlight the transformative role of AI-driven models in improving neonatal care through accurate risk assessments, timely and personalized interventions, and ultimately better survival outcomes for high-risk neonates.

**Table 6:** Studies Regarding Neonatal Outcomes and Mortality

Field of Interest	Problem Domain in Midwifery or Obstetrics	Authors	Classifiers	Number of Samples	Results
Neonatal outcomes and mortality	Predicting RDS and significant alterations in liver functions (SALV) using oxidative stress biomarkers (OSBs) and single-nucleotide polymorphisms (SNPs)	Sridharan et al., 2023 [70]	C5.0 Algorithm, Bayesian Network	Not specified	The C5.0 algorithm achieved an AUC of 0.63 for predicting SALV, with catalase identified as the most significant predictor. The Bayesian network had an AUC of 0.60 for predicting RDS, with ENOS1 being the most important predictor. Machine learning algorithms showed potential in identifying genetic markers and oxidative stress biomarkers associated with neonatal RDS and liver function alterations. Further validation in prospective studies is needed.
Neonatal outcomes and mortality	Predicting adverse neonatal outcomes in pregnancies complicated by gestational diabetes (GDM)	Houri et al., 2023 [71]	Neural Network Model, XGBoost, Keras Framework (TensorFlow)	452 participants	The model achieved an accuracy of 82% at the time of GDM diagnosis and 91% at delivery. Significant predictors included maternal age, pre-pregnancy BMI, and results from the 3-hour 100 g oral glucose tolerance test. Composite adverse outcomes included conditions such as large or small for gestational age, shoulder dystocia, low fetal umbilical pH (< 7.2), NICU admission, respiratory distress syndrome (RDS), hyperbilirubinemia, and polycythemia. The neural network model demonstrated strong predictive capability for adverse neonatal outcomes.
Neonatal outcomes and mortality	Predicting RDS in very low birth weight premature infants to guide treatment	Jang et al., 2023 [72]	Five-layer Deep Neural Network, Ensemble Model from 5-fold Cross-Validation, Machine Learning Algorithms (seven models tested)	13,087 infants across 76 hospitals	The ensemble deep neural network model achieved an AUC of 0.9187, sensitivity of 83.03%, specificity of 87.50%, and accuracy of 84.07%. A web application was developed for clinical use to help determine the necessity of surfactant treatment and improve neonatal resuscitation decisions.
Neonatal outcomes and mortality	Mortality risk prediction in very-low-birth-weight infants	Na et al., 2023 [73]	MLP-based model with ensemble ML	15,790 infants	AUCs 0.932 (TL-1d), 0.973 (TL-7d), 0.950 (TL-dc)
Neonatal outcomes and mortality	Predicting neonatal mortality in pregnant women admitted to the ICU	Espinola-Sánchez et al., 2023 [74]	Gradient Boosting	280	AUC: 0.98 (95% CI: 0.95-1); Sensitivity: 0.98 (95% CI: 0.94-1); Specificity: 0.98 (95% CI: 0.93-1); Key predictors: gestational age, eclampsia, kidney infection, maternal age, placenta complications, severe preeclampsia, prenatal checkups, and miscarriage history
Neonatal outcomes and mortality	Estimating gestational age and identifying preterm or SGA newborns	Vitral et al., 2023 [75]	Machine learning algorithm (device-based)	305	Intraclass correlation coefficient: 0.829 (95% CI: 0.785-0.863); accuracy for preterm classification: 78.4% (95% CI: 73.3-81.6); sensitivity: 96.2% (95% CI: 92.8-98.2); accuracy for SGA classification: 62.3% (95% CI: 56.6-67.8); mean GA underestimation by 2.8 days

Discussion

This systematic review aimed to explore the applications of AI and ML in obstetrics and midwifery. Based on the findings of the studies reviewed, three key findings emerge. First, there is a notable increase in the adoption of ML applications across various areas of obstetric and midwifery care, such as embryo selection, prediction of pregnancy complications, fetal monitoring, and the assessment of maternal and neonatal risks [12,21,61]. The results in these areas highlight the potential of ML in enhancing diagnostic accuracy, supporting clinical decision-making, and improving the overall quality of healthcare delivery in both obstetrics and midwifery.

Second, the present systematic review concludes that ML algorithms, including Random Forest, XGBoost, and Support Vector Machines, have the ability to outperform conventional statistical methods in predicting the development of serious adverse events, such as preeclampsia, gestational diabetes, and preterm birth [22,23,30]. This is in line with the general expectations regarding AI, which is expected to outperform traditional tools [2]. Indeed, these AI and ML boosted models demonstrated superior predictive accuracy compared to traditional approaches, with some achieving an area under the curve exceeding 0.9 [21,24]. Hence, this finding highlights their effectiveness in clinical risk assessment and early intervention planning.

Third, a significant advancement identified in the present systematic review is the use of ML to facilitate personalized interventions by generating individualized risk assessments. Personalized interventions are based on patient-specific factors, requiring related assessments. More specifically, studies that made use of interpretability tools, such as SHAP and LIME, indicate that these tools are quite helpful, empowering healthcare providers to make more informed decisions [23,26]. By adopting a personalized approach, individual parameters are considered, enabling clinicians to adjust treatment strategies according to each patient's unique risk profile, ultimately improving maternal and neonatal health outcomes [33,73].

In general, the starting point of the present study was the previous systematic review carried out by Barbounaki and Vivilaki [9]. For that reason, it is interesting to note some common findings between the two studies. More specifically, both reviews highlight the success of ML applications in improving predictions for various obstetric and midwifery outcomes, particularly in areas such as embryo selection for IVF [12,13] and predicting implantation outcomes [14]. In line with the previous findings, during the period 2022-2025 ML has also been successfully utilized for forecasting pregnancy-related complications, including preeclampsia, preterm birth, and neonatal mortality [21,22,24]. In addition, both reviews emphasize the role of AI-driven systems in enhancing fetal monitoring and reproductive medicine through the use of advanced analysis of fetal heart rate patterns and uterine contractions [60]. As a result, both systematic reviews conclude that AI holds substantial promise for improving the quality of obstetric and midwifery care through early risk detection and intervention.

However, the recent review introduces significant advancements not discussed in the earlier work by Barbounaki and Vivilaki [9], which could be attributed to the improvement of AI and ML over time. One notable development that occurred during the previous years is the broader range of AI applications. More specifically, there are significant advancements, since real-time fetal health monitoring is available, using advanced imaging tools such as MRI and ultrasound-based AI models. These models assist in detecting fetal abnormalities, such as cleft lip and Down syndrome [62,63]. Furthermore, the review highlights the use of cutting-edge deep learning algorithms, such as EfficientNetB0 and Vision Transformer, which have achieved higher accuracy rates in predicting adverse maternal outcomes [32]. A particularly novel finding is the incorporation of explainable AI techniques like SHAP and LIME, which provide clearer interpretations of risk factors, thereby improving clinician-patient trust and facilitating transparent clinical decisions [23,26]. Finally, the inclusion of larger, multicentric datasets and external validations in these new studies ensures a higher degree of generalizability for AI models [14,74], improving upon earlier research that lacked extensive validation. These innovations mark a substantial evolution in AI applications since the findings of Babounaki and Vivilaki’s [9] initial review, leading to the conclusion that significant progress has been made in recent years.

Based on all the above, AI and ML should play a pivotal role in obstetrics and midwifery, offering innovative approaches to enhance patient care and improve clinical outcomes. These technologies are being used for tasks such as selecting embryos during IVF, predicting complications during pregnancy, monitoring fetal development, and managing labor and delivery more effectively. Through the analysis of various data, ranging from ultrasound images to electronic health records, AI and ML models can identify patterns that may not be visible to the human eye. This enables healthcare professionals to detect high-risk pregnancies earlier, customize treatment plans tailored to individual patients, and improve decision-making during childbirth. In addition, AI can automate complex tasks, like tracking fetal heart rates or identifying congenital abnormalities through imaging, which not only saves time but also improves diagnostic accuracy, ultimately leading to better outcomes. Therefore, it is crucial to adapt AI and ML in clinical care.

The potential of AI and ML in obstetrics and midwifery is promising, but several significant challenges must be addressed in order to foster their adoption in everyday clinical practice. A key obstacle is the absence of standardized guidelines for incorporating AI and ML into routine care, which creates uncertainty about how to use these tools most effectively. Another concern is that many AI models are trained on limited or non-diverse datasets, raising questions about their reliability and accuracy across varied patient populations. Additionally, algorithmic bias remains a pressing issue, along with the challenge of many AI systems functioning as “black boxes,” which leaves clinicians uncertain about how decisions are made. However, it is important to acknowledge the methodological heterogeneity among the included studies in our research. The included studies varied in terms of AI methodologies, clinical applications, and outcome measures, which poses challenges for drawing direct comparisons. These differences underline the necessity for standardized guidelines and validation frameworks to ensure the consistency, reliability, and applicability of AI tools in diverse clinical settings. In order to address those concerns, future research should focus on building larger, more diverse datasets that better represent different patient demographics and healthcare settings. Additionally, healthcare professionals must receive continuous training to understand, implement, and make the most of AI and ML tools, ensuring that they can effectively integrate them into their clinical practices and enhance patient care. Finally, the development of AI tools supported by ML will require significant collaboration between developers and healthcare professionals.

## Conclusions

This study highlights the transformative potential of AI and ML in obstetrics and midwifery. The present systematic review emphasizes the increasing integration of AI and ML across various domains, including embryo selection, prediction of pregnancy complications, fetal monitoring, and risk assessment for both maternal and neonatal outcomes. Notably, recent advancements have demonstrated superior predictive performance compared to traditional methods applied in obstetrics and midwifery, enabling earlier detection of complications and enhancing clinical decision-making. A key trend, identified by this systematic review, is the application of ML for personalized interventions. However, several limitations still exist, such as the lack of standardized clinical integration and potential algorithmic biases. Moving forward, it is crucial to focus on expanding datasets, improving model interpretability, and fostering collaborations between healthcare professionals, professionals involved in the development of ML powered AI applications and decision makers. Overall, the substantial number of studies reporting significant effects suggests that the time for large-scale AI and ML adoption in obstetrics and midwifery has arrived.
